# Fixed-dose combination antihypertensive therapy and healthcare utilization in U.S. adults with hypertension: a propensity score–based analysis of a nationally representative population

**DOI:** 10.3389/fphar.2026.1787754

**Published:** 2026-03-06

**Authors:** Eissa A. Jafari

**Affiliations:** 1 Department of Pharmacy Practice, College of Pharmacy, Jazan University, Jazan, Saudi Arabia; 2 Pharmacy Practice Research Unit, College of Pharmacy, Jazan University, Jazan, Saudi Arabia

**Keywords:** antihypertensives, fixed-dose combination, healthcare utilization, hypertension, Medical Expenditure Panel Survey

## Abstract

**Background:**

Fixed-dose combination (FDC) antihypertensive therapy is recommended by contemporary guidelines to improve adherence and blood pressure control. However, real-world evidence evaluating its impact on healthcare utilization, compared with the multi-pill combination (MPC) therapy in hypertension (HTN) patients, remains limited. This study compared healthcare utilization among US adults with HTN receiving FDC versus MPC therapy.

**Methods:**

We conducted a cross-sectional study on nationally representative data from the Medical Expenditure Panel Survey (2013-2022). Adults aged ≥18 years with a diagnosed HTN on ≥2 antihypertensive classes were classified as FDC or MPC users. Inverse probability of treatment weighting was applied to balance covariates. Weighted negative binomial regression models were used to assess the impact of FDC versus MPC on healthcare utilization, including office-based visits, outpatient visits, emergency department visits, hospitalizations, and prescription fills. A 1:1 propensity score matching (PSM) analysis was conducted as a sensitivity analysis to assess findings robustness.

**Results:**

Among 18,269 adults receiving ≥2 antihypertensive therapies, 5,849 were FDC users, and 12,420 were MPC users. Compared with MPC users, FDC users had significantly lower emergency department visits (rate ratio [RR] = 0.712; 95% confidence interval [CI]: 0.636–0.796; p < 0.0001), hospitalizations (RR = 0.721; 95% CI: 0.545–0.953; p = 0.0219), office-based visits (RR = 0.934; 95% CI: 0.879–0.993; p 0.0281), and prescription fills (RR = 0.853; 95% CI: 0.815–0.893; p < 0.0001). No significant difference was observed in the outpatient visit rate. Findings were consistent in PSM analysis.

**Conclusion:**

FDC antihypertensive therapy was associated with significantly lower acute care utilization and prescription burden while preserving routine outpatient care, compared with the MPC therapy. These findings support FDC therapy use as a high-value strategy to enhance real-world HTN management and reduce acute healthcare utilization.

## 1 Introduction

Hypertension (HTN) remains the leading cause of stroke, heart disease, and premature mortality in the United States (US) (Martin et al., 2024; Wu et al., 2025). Recent data from the National Health and Nutrition Examination Survey indicated that nearly half of the US adults have HTN, yet fewer than half of those with HTN achieve the recommended blood pressure control target (Fryar et al., 2023). Economically, HTN ranks among the most burdensome chronic conditions in the US, owing to its high prevalence, lifelong treatment requirements, and associated cardiovascular complications (Kirkland et al., 2018; Zhang et al., 2017). The combination of high prevalence, chronic pharmacotherapy requirements, and cardiovascular morbidity contributes to substantial healthcare utilization, including emergency department visits, hospitalizations, and prescription drug use (Kirkland et al., 2018; Zhang et al., 2017; Singh and Yu, 2016). Estimates from nationally representative data suggested that individuals with HTN incur roughly $1,500–2,500 more in annual medical expenditures than adults without HTN, contributing to over $100 billion in HTN-related medical spending each year (Kirkland et al., 2018; Zhang et al., 2017).

The 2017 American College of Cardiology/American Heart Association (ACC/AHA) HTN guideline recommends initiating combination pharmacotherapy with two antihypertensive agents from different classes for most adults with stage 2 HTN and patients with stage 1 HTN who have elevated atherosclerotic cardiovascular disease risk (Whelton et al., 2017). These patients may also require subsequent treatment intensification that involves adding agents from different classes to achieve adequate blood pressure control. As a result, a large proportion of adults with HTN are prescribed a multidrug regimen, often taken as separate pills (Whelton et al., 2017). However, the clinical effectiveness of such a regimen is often compromised by medication nonadherence (Verma et al., 2018; Parati et al., 2021), with evidence indicating that approximately one-third of adults with HTN do not adequately adhere to their prescribed treatment (Chang et al., 2015). This issue is multifactorial, driven by a complex regimen that requires multiple daily doses, high pill burden, forgetfulness, treatment fatigue, medication costs, and a perceived lack of symptoms or disease severity (Vawter et al., 2008; Zhou et al., 2024). Nonadherence is associated with significantly increased risks of stroke, myocardial infarction (MI), heart failure (HF), and cardiovascular mortality (Kim et al., 2025; Lee et al., 2017). Additionally, poor blood pressure control resulting from medication nonadherence may increase healthcare utilization, including emergency department visits and hospitalizations, thereby escalating healthcare costs (Pittman et al., 2010; Lee et al., 2024). These challenges highlight the need for therapeutic strategies that simplify regimens while maintaining or improving clinical effectiveness.

Fixed-dose combination (FDC) therapy, which co-formulates two or more antihypertensive agents in a single pill, has emerged as a promising strategy to address these challenges. By simplifying treatment regimens and reducing pill burden, FDC has been shown to significantly improve medication adherence, compared with a multi-pill regimen (Sarzani et al., 2022). A population-based retrospective cohort study by Verma et al. demonstrated that patients initiated on FDC therapy had a greater proportion of days covered with medication (median 70%) than those on multi-pill combinations (median 42%) (Verma et al., 2018). In the same study, FDC users experienced lower rates of adverse clinical events, death, or hospitalization due to MI, HF, or stroke (Verma et al., 2018; Snyman et al., 2024). These findings have been supported by meta-analyses and systematic reviews showing that FDC therapy not only improves BP control but also reduces the incidence of major cardiovascular events (Kawalec et al., 2018; Kengne et al., 2024; Tsioufis et al., 2020).

Beyond improving adherence and clinical outcomes, FDC therapy may also reduce healthcare utilization. However, much of the existing evidence comes from small cohorts, integrated health systems, or payer-specific datasets such as Medicaid or Medicare. These studies are often limited by non-representative populations, a lack of longitudinal data, or a narrow focus on a single utilization outcome (e.g., hospitalization or emergency department visits) (Hess et al., 2008; Yang et al., 2010; Zhang et al., 2025). As a result, there is limited evidence on the broader impact of FDC therapy on healthcare utilization in the general US HTN population. The Medical Expenditure Panel Survey (MEPS) presents a unique opportunity to fill this gap, as it provides nationally representative data on the US civilian noninstitutionalized population, along with detailed information on medical conditions, prescription drug use, and multiple categories of healthcare utilization. Therefore, the objective of this study was to compare healthcare utilization, including office-based visits, hospital outpatient visits, emergency department visits, prescription fills, and hospitalizations in adults with HTN treated with FDC versus multi-pill combination (MPC) therapy in MEPS from 2013 to 2022. By doing so, this study aimed to generate updated, generalizable insights into the real-world impact of FDC therapy on healthcare utilization in US adults with HTN, thereby informing both clinical decision-making and health policy strategies to optimize HTN management.

## 2 Methods

### 2.1 Data source

This study used publicly available data from the MEPS Household Component (HC) spanning the years 2013–2022. MEPS is a nationally representative survey of the US civilian noninstitutionalized population that collects detailed information on demographics, medical conditions, healthcare utilization, prescription drug use, and medical expenditures. The survey employs a complex multistage probability sampling design, incorporating clustering, stratification, and person-level sampling weights to generate national estimates. The Full-Year Consolidated, Medical Conditions, and Prescribed Medicines files were used in this analysis and were linked using the unique person identifier (DUPERSID).

MEPS collects healthcare utilization and prescription data through the HC, which captures event-level use and expenditures for office-based and hospital-based care, as well as prescribed medicines, during household interview rounds. Annual utilization measures in the MEPS full-year consolidated file are constructed by summing events across the rounds that cover the given survey year. In addition, MEPS conducts the Medical Provider Component (MPC), a follow-back survey of medical providers and pharmacies used by respondents; MPC data are used to improve the quality of MEPS data collection.

### 2.2 Study population

We included adults aged ≥18 years with a diagnosis of HTN, identified by the International Classification of Diseases, Ninth and Tenth Revision (ICD-9/10) codes from the MEPS Medical Conditions file. To ensure active antihypertensive treatment and minimize misclassification of short-term or incidental use, patients were required to be prescribed at least two different antihypertensive drug classes, with a minimum of two prescription fills during the same survey year. Individuals with pregnancy or those treated with only a single antihypertensive class were excluded. Eligible patients were classified into two mutually exclusive treatment groups based on their antihypertensive therapy: FDC antihypertensive users and MPC users. FDC therapy was defined as the use of a single-pill product containing ≥2 antihypertensive drug classes, while MPC therapy was defined as the use of ≥2 antihypertensive drug classes dispensed as separate pills during the same survey year. FDC and MPC antihypertensives were defined based on Multum Lexicon therapeutic classification codes from the Prescribed Medicines files (Supplementary Table S1). The primary exposure variable was a binary indicator denoting whether a patient received FDC or MPC therapy.

### 2.3 Covariates

The study included a comprehensive set of demographic, socioeconomic, behavioral, and clinical variables. Demographic variables included age (18-39, 40-64, and ≥65 years), sex (male or female), and race/ethnicity (Hispanic, non-Hispanic White, non-Hispanic Black, non-Hispanic Asian, and other or multiracial groups). Socioeconomic factors included educational attainment (no degree, high school diploma, some college or associate degree, or college degree and above), marital status (married; unmarried; divorced, widowed, or separated), insurance type (private, public, or uninsured), and household income level (poor, near-poor, low, middle, or high income). Geographic variation was captured using US census regions (Northeast, Midwest, South, and West).

Behavioral variables included current smoking status and regular physical activity, defined as engaging in at least 30 min of moderate-to-vigorous physical activity at least 5 days per week. Clinical comorbidities were identified using ICD-9/10 codes and included dyslipidemia, chronic kidney disease (CKD), diabetes, stroke, MI, HF, coronary heart disease (CHD), chronic obstructive pulmonary disease (COPD), asthma, Alzheimer’s disease and related dementias (ADRD), osteoarthritis, gastroesophageal reflux disease (GERD), anxiety, and depression.

### 2.4 Study outcomes

Healthcare utilization outcomes were measured as annual counts within the same survey year as treatment classification, including the number of office-based visits, hospital outpatient visits, emergency department visits, prescription fills, and inpatient hospitalizations.

### 2.5 Statistical analysis

Descriptive analyses were conducted to compare patient characteristics between FDC and MPC users. Categorical variables were summarized using frequencies and weighted percentages, while continuous variables were reported as means with standard error. Between-group differences were assessed using absolute standardized mean differences (SMDs), with values < 0.10 considered indicative of adequate covariate balance.

To address potential confounding due to non-random treatment selection, propensity scores (PS) representing the probability of receiving FDC therapy were estimated using a multivariable logistic regression model that included demographic, socioeconomic, behavioral, and clinically relevant covariates. Stabilized inverse probability of treatment weights (IPTW) were derived from the estimated PS and truncated at the 95th percentile to mitigate the influence of extreme weights. Final analytic weights were generated by multiplying the stabilized IPTW by the MEPS full-year person-level survey weights, thereby accounting for both treatment selection and complex survey design. Covariate balance after weighting was reassessed using SMDs, and the combined weight was applied in all subsequent analyses.

Associations between FDC therapy and healthcare utilization outcomes were evaluated using survey-weighted negative binomial regression models, selected based on evidence of overdispersion in the outcome distributions. Separate models were fitted for each utilization outcome. Results were reported as rate ratios (RRs) with corresponding 95% confidence intervals (CIs) and p-values.

To complement the IPTW approach and assess the robustness of our findings, we conducted a sensitivity analysis using 1:1 propensity score matching (PSM). Nearest-neighbor matching was performed without replacement on the logit of the estimated PS, using a caliper width of 0.2 standard deviations. The same set of covariates included in the IPTW model was used for matching. Covariate balance in the matched cohort was reassessed using SMDs. Survey-weighted negative binomial regression models were then applied to the matched sample to estimate the associations between FDC use and healthcare utilization outcomes. The results from the matched analysis were also reported as RR with 95% CIs and p-values.

All statistical analyses accounted for MEPS survey weights, strata, and primary sampling units. Analyses were performed using SAS version 9.4 (SAS Institute, Cary, NC) and R version 4.3.3 (R Foundation for Statistical Computing, Vienna, Austria).

## 3 Results

### 3.1 Data preparation and cohort selection

**FIGURE 1:**
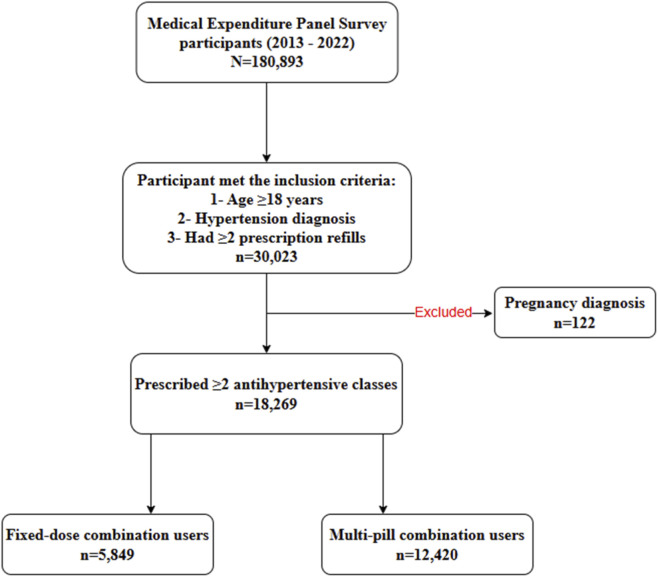
Flowchart of data preparation and cohort selection.

Among 180,893 MEPS participants surveyed between 2013 and 2022, a total of 30,023 adults aged ≥18 years were identified with diagnosed HTN and at least two antihypertensive prescription refills. After excluding 122 individuals with pregnancy-related diagnoses, 29,901 participants remained eligible. Of these, 18,269 individuals were prescribed two or more antihypertensive drug classes, indicating use of combination therapy. Within this population, 5,849 participants were classified as FDC users and 12,420 as MPC users (Figure 1).

### 3.2 Patient characteristics

Table 1 summarizes the patient characteristics of the overall cohort and stratified by treatment group (FDC vs. MPC), before and after IPTW. The mean age of the cohort was 66 years, with the majority aged ≥65 years (57%). The sex distribution was balanced, and the majority of participants were non-Hispanic White (69%), followed by non-Hispanic Black (15%) and Hispanic (9%). Common comorbidities included dyslipidemia (72%), diabetes (33%), osteoarthritis (32%), and CHD (21%).

**TABLE 1:** Patient characteristics.

​	Total population	Before IPTW			After IPTW		
Characteristic	Overall N = 18,269 (wt%)	FDC n = 5,849 (wt%)	MPC n = 12,420 (wt%)	SMD	FDC n = 5,849 (wt%)	MPC n = 12,420 (wt%)	SMD
Age (years)	66 (0.15)	64 (0.22)	67 (0.18)	0.240	66 (0.22)	66 (0.18)	0.022
Age category							
18–39	554 (3)	216 (4)	338 (3)	0.055	176 (3)	378 (3)	0.002
40–64	7,454 (40)	2,766 (47)	4,688 (38)	0.194	2,347 (40)	5,051 (41)	0.011
≥65	10,261 (57)	2,867 (49)	7,394 (59)	0.212	3,326 (57)	6,991 (56)	0.012
Sex							
Female	8,206 (49)	2,421 (41)	5,785 (47)	0.105	2,635 (45)	5,578 (45)	0.003
Male	10,063 (51)	3,428 (59)	6,635 (53)	0.105	3,214 (55)	6,842 (55)	0.003
Race/Ethnicity							
Hispanic	2,605 (9)	818 (14)	1,787 (14)	0.012	838 (14)	1,768 (14)	0.003
Non-hispanic white	9,830 (69)	2,922 (50)	6,908 (56)	0.114	3,130 (54)	6,686 (54)	0.006
Non-hispanic black	4,560 (15)	1,716 (29)	2,844 (23)	0.147	1,474 (25)	3,100 (25)	0.005
Non-hispanic asian	790 (4)	270 (5)	520 (4)	0.021	254 (4)	539 (4)	0.000
Other races	484 (3)	123 (2)	361 (3)	0.051	153 (3)	327 (3)	0.001
Education							
No degree	3,547 (13)	1,036 (18)	2,511 (20)	0.064	1,156 (20)	2,418 (20)	0.008
High school diploma	8,425 (46)	2,665 (46)	5,760 (46)	0.016	2,703 (46)	5,724 (46)	0.002
Some college/associate degree	2,312 (15)	828 (14)	1,484 (12)	0.066	737 (13)	1,571 (13)	0.001
Bachelor’s degree/higher education	3,985 (26)	1,320 (22)	2,665 (22)	0.027	1,253 (21)	2,707 (21)	0.009
Insurance							
Private	9,458 (61)	3,440 (59)	6,018 (48)	0.209	2,988 (51)	6,421 (52)	0.012
Public	8,217 (37)	2,161 (37)	6,056 (49)	0.240	2,675 (46)	5,596 (45)	0.013
Uninsured	594 (2)	248 (4)	346 (3)	0.079	186 (3)	403 (3)	0.003
Poverty category							
Poor/negative	3,341 (12)	937 (16)	2,404 (19)	0.088	1,019 (17)	2,338 (18)	0.037
Near poor	1,204 (5)	345 (6)	859 (7)	0.042	398 (7)	835 (7)	0.003
Low income	2,968 (14)	857 (15)	2,111 (17)	0.064	907 (16)	2,072 (17)	0.032
Middle income	4,996 (28)	1,642 (28)	3,354 (27)	0.024	1,610 (28)	3,386 (27)	0.006
High income	5,760 (41)	2,068 (35)	3,692 (30)	0.120	1,915 (32)	3,789 (31)	0.048
Marital status							
Married	8,955 (56)	3,075 (53)	5,880 (47)	0.105	3,027 (52)	5,925 (48)	0.081
Divorced/widowed/separated	7,176 (35)	2,109 (36)	5,067 (41)	0.098	2,225 (38)	4,970 (40)	0.040
Never married	2,138 (9)	665 (11)	1,473 (12)	0.015	597 (10)	1,525 (12)	0.065
Region							
Northeast	3,056 (17)	918 (16)	2,138 (17)	0.041	975 (17)	2,077 (17)	0.001
Midwest	3,782 (23)	1,183 (20)	2,599 (21)	0.017	1,210 (21)	2,573 (21)	0.001
South	7,920 (42)	2,797 (47)	5,123 (41)	0.133	2,538 (43)	5,385 (43)	0.001
West	3,511 (18)	951 (16)	2,560 (21)	0.112	1,126 (19)	2,385 (19)	0.001
Physical exercise	7,104 (40)	2,377 (41)	4,727 (38)	0.053	2,263 (39)	4,823 (39)	0.003
Smoking status	11,567 (64)	3,977 (68)	7,590 (61)	0.144	3,706 (63)	7,862 (63)	0.001
Dyslipidemia	13,119 (72)	3,930 (67)	9,189 (74)	0.150	4,208 (72)	8,923 (72)	0.002
CKD	505 (3)	60 (1)	445 (3)	0.171	213 (4)	345 (3)	0.049
Diabetes	6,645 (33)	1,869 (32)	4,776 (39)	0.136	2,162 (37)	4,526 (36)	0.011
Stroke	2,527 (13)	542 (9)	1,985 (16)	0.203	829 (14)	1,723 (14)	0.009
MI	2,549 (14)	414 (7)	2,135 (17)	0.313	877 (15)	1,739 (14)	0.028
HF	770 (4)	85 (2)	685 (6)	0.223	274 (5)	525 (4)	0.022
CHD	3,724 (21)	617 (11)	3,107 (25)	0.385	1,239 (21)	2,539 (20)	0.018
COPD	2,623 (14)	700 (12)	1,923 (16)	0.102	804 (14)	1,813 (15)	0.024
Asthma	2,969 (15)	883 (15)	2,086 (17)	0.046	934 (16)	2,050 (17)	0.015
ADRD	2,712 (13)	643 (11)	2,069 (17)	0.165	811 (14)	1,930 (16)	0.047
Osteoarthritis	5,820 (32)	1,708 (29)	4,112 (33)	0.084	1,849 (32)	4,030 (32)	0.018
GERD	3,599 (19)	1,042 (18)	2,557 (21)	0.070	1,166 (20)	2,497 (20)	0.004
Anxiety	2,920 (16)	874 (15)	2,046 (17)	0.042	905 (16)	2,037 (16)	0.026
Depression	1,980 (11)	516 (9)	1,464 (12)	0.098	567 (10)	1,418 (11)	0.056

Abbreviation: FDC; fixed-dose combination, MPC; multi-pill combination, IPTW; inverse probability of treatment weighting, SMD; standardized mean difference, ADRD; Alzheimer’s disease and related dementia, GERD; gastroesophageal reflux disease, COPD; chronic obstructive pulmonary disease; CKD; chronic kidney disease, CHD; coronary heart disease, HF; heart failure, MI; myocardial infarction, wt%; weighted percentage.

**FIGURE 2:**
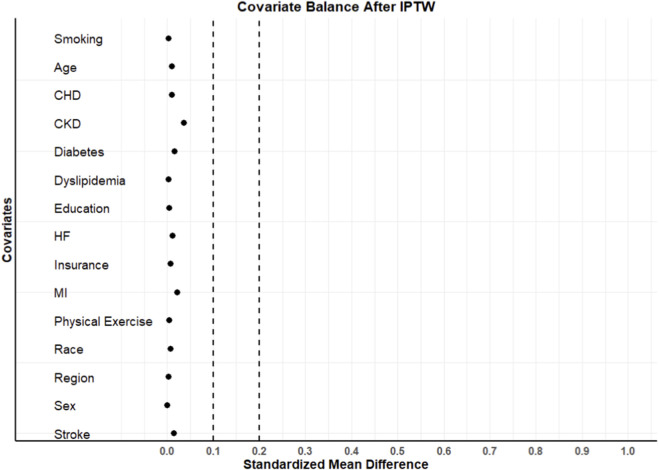
Covariates balance after IPTW. Abbreviation: IPTW; Inverse probability of treatment weighting, CKD; Chronic kidney disease, CHD; Coronary heart disease, HF; Heart failure, MI; Myocardial infarction.

Before weighting, many covariates were imbalanced between FDC and MPC. However, after applying IPTW and PSM, almost all characteristics were well balanced between the FDC and MPC groups, with SMD below the threshold of 0.1, indicating good covariate balance (Table 1; Figure 2; Supplementary Figure S1). The distribution and overlap of propensity scores between treatment groups are shown in Supplementary Figure S2.

### 3.3 Impact of FDC on healthcare utilization

Unadjusted comparisons of healthcare utilization are summarized in Table 2. Across utilization categories, MPC users showed significantly higher healthcare utilization than FDC users (all p < 0.0001).

**TABLE 2:** Comparison of healthcare utilization between FDC and MPC.

Healthcare utilization category	FDC N = 5,849	MPC N = 12,420	​
	Mean (SE)	Mean (SE)	P-value
Office-based physician visits	5.97 (0.12)	7.32 (0.10)	<0.0001
Outpatient physician visits	0.55 (0.04)	0.74 (0.04)	<0.0001
Prescription fills	28.77 (0.49)	38.92 (0.49)	<0.0001
Emergency department visits	0.25 (0.01)	0.44 (0.01)	<0.0001
Hospitalization	0.76 (0.07)	1.63 (0.09)	<0.0001

Abbreviation: FDC; fixed-dose combination, MPC; multi-pill combination, SE; standard error.

Adjusted associations between antihypertensive regimen and healthcare utilization are presented in Table 3. In IPTW negative binomial regression models, FDC use was associated with significantly lower healthcare utilization across most outcomes, compared with MPC use. Specifically, FDC users had lower rates of office-based visits (RR = 0.934; 95% CI: 0.879–0.993; p = 0.0281), prescription fills (RR = 0.853; 95% CI: 0.815–0.893; p < 0.0001), emergency department visits (RR = 0.712; 95% CI: 0.636–0.796; p < 0.0001) and hospitalizations (RR = 0.721; 95% CI: 0.545–0.953; p = 0.0219). FDC therapy was not associated with a significant reduction in outpatient visits, compared to MPC therapy (RR = 0.917; 95% CI: 0.731–1.151; p = 0.4552). Similar results were observed in the adjusted multivariate and PSM analyses (Table 3; Table 4).

**TABLE 3:** Results of adjusted multivariate and IPTW models evaluating the association of FDC versus MPC with healthcare utilization.

Outcome	adjusted multivariate model			IPTW model		
	RR	95% CI	P-value	RR	95% CI	P-value
Office-based visits	0.915	(0.875–0.956)	<0.0001	0.934	(0.879–0.993)	0.0281
Outpatient visits	0.864	(0.710–1.052)	0.1459	0.917	(0.731–1.151)	0.4552
Prescription fills	0.830	(0.800–0.860)	<0.0001	0.853	(0.815–0.893)	<0.0001
Emergency department visits	0.705	(0.638–0.779)	<0.0001	0.712	(0.636–0.796)	<0.0001
Hospitalization	0.666	(0.545–0.814)	<0.0001	0.721	(0.545–0.953)	0.0219

Abbreviation: FDC; fixed-dose combination, MPC; multi-pill combination, IPTW; inverse probability of treatment weighting, RR; relative ratio, CI; confidence interval.

**TABLE 4:** Results of the PSM model evaluating the association of FDC versus MPC with healthcare utilization.

Outcome	PSM model N = 10,054, 5,027 each arm		
	RR	95% CI	P-value
Office-based visits	0.905	(0.849–0.965)	0.0022
Outpatient visits	0.877	(0.697–1.103)	0.2605
Prescription fills	0.826	(0.786–0.867)	<0.0001
Emergency department visits	0.759	(0.675–0.853)	<0.0001
Hospitalization	0.760	(0.593–0.976)	0.0314

Abbreviation: FDC; fixed-dose combination, MPC; multi-pill combination, PSM; propensity score matching, RR; relative ratio, CI; confidence interval

## 4 Discussion

Despite strong guideline support for FDC therapy in HTN management, real-world evidence on its impact on healthcare utilization, compared with the MPC regimen, remains limited. This study aimed to fill this gap by evaluating healthcare utilization associated with FDC versus MPC therapy among US HTN adults requiring multidrug antihypertensive therapy. In this nationally representative analysis of US adults with HTN, we found that FDC therapy was associated with significantly lower healthcare utilization across multiple domains, including office-based visits, emergency department visits, prescription fills, and hospitalizations, compared with the MPC therapy. Importantly, FDC use was not associated with a significant reduction in outpatient visits. These findings suggest that FDC use may be associated with lower acute care utilization and overall treatment burden, while maintaining continuity of routine care, supporting further evaluation of FDC therapy as a strategy to improve the efficiency of HTN management at the population level.

Recent real-world evidence and contemporary international hypertension guidelines increasingly support early combination therapy and favor FDC for many HTN patients, largely due to better adherence and lower rates of cardiovascular events associated with it (Coca et al., 2025; Mancia et al., 2023; McEvoy et al., 2024; Writing Committee et al., 2025). In the context of prior evidence, our findings are consistent with and extend the existing literature on the benefits of FDC therapy in HTN patients (Verma et al., 2018; Hess et al., 2008; Yang et al., 2010; Zhang et al., 2025). In our MEPS-based analysis, FDC use was associated with about 15% lower rate of emergency department visits and a 29% lower rate of hospitalization, compared with MPC therapy. Similarly, in US claims analyses, patients who were prescribed FDC therapy had a 26% lower annual probability of emergency department visits and about 21% lower rate of hospitalization, compared with those prescribed free combinations (Hess et al., 2008). More recently, among Medicaid beneficiaries, FDC use was associated with 220 fewer emergency department visits and 153 fewer hospitalizations per 1,000 individuals over 1 year, compared with those on multi-pill regimens (Zhang et al., 2025). Our study adds some important dimensions to the FDC literature in HTN. First, unlike most prior US studies, which have focused on specific payer datasets, this study used MEPS data to provide a nationally representative analysis of the US hypertensive population, enhancing the generalizability beyond a single payer or healthcare system (Hess et al., 2008; Yang et al., 2010; Zhang et al., 2025). Second, the study simultaneously examined a broad spectrum of healthcare utilization outcomes, including office-based visits, hospital outpatient visits, emergency department visits, hospitalizations, and total prescription fills, rather than focusing solely on hospitalizations or emergency department visits, thereby offering a more comprehensive assessment of how FDC therapy influences overall patterns of care. Third, the use of both IPTW and PSM provides robust adjustment for observed confounding and improves the credibility of the estimated associations between FDC use and healthcare utilization. Together, these features position this study as a complementary contribution to the existing FDC evidence.

The observed reduction in prescription fills among FDC users is best interpreted as a result of regimen consolidation rather than a reduction in pharmacological intensity. By design, FDC products combine two or more antihypertensive agents into a single pill, allowing patients to achieve comparable therapeutic coverage with fewer prescriptions and refill events. Prior cohort studies and meta-analyses have shown that FDC was associated with higher adherence, higher medication possession ratios, and greater persistence than a free-equivalent multipill regimen (Verma et al., 2018; Tung et al., 2015; Sherrill et al., 2011; Du et al., 2018). In our study, the fewer prescription fills in the FDC group in MEPS likely reflect the intended simplification of therapy, reduced pill burden, and fewer pharmacy visits, rather than systematically lower exposure to antihypertensive drugs.​ Furthermore, because prescription fills in MEPS capture total medication use and not antihypertensives alone, the observed reduction may also reflect downstream effects of improved HTN control, such as fewer prescriptions related to cardiovascular complications or symptom management, resulting in an overall lower pharmacological burden (Verma et al., 2018).

The observed reductions in both emergency department visits and hospitalizations among FDC users are clinically plausible and mechanistically consistent with the established benefits of FDC in HTN management. The fewer emergency department visits are consistent with evidence showing that better adherence and more consistent blood pressure control associated with FDC reduce acute decompensations such as hypertensive urgencies, strokes, and HF exacerbations, which frequently present through the ED (Lee et al., 2024; Yang et al., 2016). The parallel reduction in hospitalizations fits the same pathway; sustained blood pressure control achieved through FDC is expected to lower the incidence of severe complications like stroke, MI, and decompensated HF that require inpatient admissions (Verma et al., 2018; Kawalec et al., 2018; Benjamin et al., 2019). Although MEPS lacks direct clinical data on blood pressure or cardiovascular events, the lower rates of emergency department visits and hospitalizations observed among FDC users mirror findings from prior studies, which have demonstrated a reduced risk of cardiovascular events, hospital admissions, and all-cause mortality among FDC users (Lee et al., 2024; Yang et al., 2010; Zhang et al., 2025). Furthermore, because MEPS captures all-cause hospitalizations rather than only cardiovascular events, the observed association between FDC use and fewer hospitalizations may indicate benefits that are not limited to cardiovascular causes, although the specific reasons for hospitalization cannot be determined from this dataset (Schmieder et al., 2023; Wilke et al., 2022).

In contrast to acute care settings, the outpatient visit rate was not significantly reduced among FDC users compared with MPC users. This pattern is favorable because a uniform reduction in all types of healthcare encounters, including routine ambulatory care, could raise a concern about underutilization or disengagement from follow-up or routine check-ups, rather than true improvement in disease control. Instead, the preserved rates of outpatient visits indicate that FDC users remained engaged in guideline-recommended chronic disease monitoring and management, including blood pressure assessment, medication adjustment, and comorbidity management. From a health-services perspective, this combination of outcomes is desirable: acute, and potentially avoidable events are minimized, while essential and preventive care is maintained.

It is important to recognize that these interpretations relied on inferred pathways. MEPS does not provide blood pressure measurements, detailed cardiovascular diagnoses linked to each encounter, or direct adherence metrics, so the link between FDC use and adherence, BP control, and event prevention cannot be formally tested within this dataset. Nevertheless, the consistency between the utilization patterns observed here and the ones reported in prior studies strengthens the argument that FDC therapy is associated with more favorable real-world healthcare utilization outcomes among patients with HTN requiring combination therapy (Verma et al., 2018; Hess et al., 2008; Yang et al., 2010; Zhang et al., 2025).

The findings from our study highlight the potential value of FDC therapy as a strategy to improve the efficiency of HTN management while reducing acute healthcare utilization without compromising routine ambulatory care. The FDC benefits observed in this study are particularly relevant for patients with high comorbidity burden or socioeconomic barriers to adherence, in whom simplifying therapy may have a greater impact (Sarzani et al., 2022; Munoz et al., 2019). Clinicians may consider FDC therapy not only for their pharmacological equivalence but also for their potential association with lower healthcare utilization.

From a policy perspective, these results support broader access to FDC therapy. Healthcare systems and payers should explore strategies to reduce barriers and expand access to FDC therapy, particularly in under-resourced settings. Additionally, targeted interventions to educate providers about the clinical and economic benefits of FDCs could help bridge the gap between evidence and prescribing behavior. Future research should build on these findings by examining differences among specific FDCs and their respective clinical outcomes, which would help identify the most effective regimen for real-world use. Moreover, qualitative research exploring patient and provider perspectives on FDC use could provide valuable insights into implementation barriers and inform more patient-centered prescribing practices.

Our study is not without limitations. First, while the MEPS includes pharmacy-validated and detailed prescription records, medication fills do not guarantee adherence. As such, the observed associations may reflect prescribed therapies rather than medications truly taken as directed. Because MEPS relies on household reporting, utilization and medication exposure may be affected by recall bias and misclassification. Although the provider and pharmacy validate the accuracy and completeness, some underreporting and discrepancies may persist. Second, although IPTW, PSM, and adjustment for a comprehensive set of demographic, socioeconomic, behavioral, and clinically relevant variables were applied, residual confounding cannot be ruled out due to unmeasured factors such as baseline blood pressure level and hypertension severity and duration, medication adherence, prior healthcare utilization, prescriber preference, and formulary coverage. Third, blood pressure measurements, adherence metrics, and adjudicated cardiovascular events are not available, making it challenging to directly validate the proposed mechanism linking FDC therapy with reduced acute care utilization. Instead, our interpretations relied on supporting evidence from previous literature. Fourth, the exposure classification used in this study was the overall use of FDC or MPC regimen, and did not account for variation in drug classes, dosages, or specific combinations, which may mask clinical differences between antihypertensive classes. Fifth, temporality cannot be established in this study because treatment classification and utilization outcomes were measured within the same MEPS survey year. Some utilization events may have occurred before treatment classification, so findings should be interpreted as associations, not causal effects. Sixth, survey year was not adjusted for in the PS model; if FDC uptake or healthcare utilization patterns changed over 2013–2022, residual confounding by time is possible. Lastly, treatment switching between FDC and MPC regimens may have occurred within calendar years, but the lack of precise prescription timing in MEPS limits detection of such changes and may introduce exposure misclassification. Despite these limitations, this study provided robust, nationally representative evidence on the association between antihypertensive treatment strategies and healthcare utilization in the US HTN population.

## 5 Conclusion

In this nationally representative cohort of US adults with HTN requiring combination therapy, FDC was associated with significantly lower healthcare utilization across multiple domains, including emergency department visits, hospitalizations, and prescription fills, while preserving routine outpatient care. Clinicians may consider FDC as a strategy to enhance real-world treatment effectiveness, not just as pharmacologic alternatives. Future research should explore the comparative effectiveness of specific FDCs, evaluate patient and provider perspectives on FDCs, and identify implementation strategies that could bridge the gaps between evidence and prescribing practices.

## Data Availability

The data used in this study are publicly available from the Medical Expenditure Panel Survey, sponsored by the Agency for Healthcare Research and Quality, and can be accessed at: Medical Expenditure Panel Survey Home. All results relevant to this study are included in the article and are available upon reasonable requests to the corresponding author.
