# Supplementary material for: Fixed-dose combination antihypertensive therapy and healthcare utilization in U.S. adults with hypertension: a propensity score–based analysis of a nationally representative population
Source: Front Pharmacol. 2026 Mar 6;17:1787754. doi: 10.3389/fphar.2026.1787754 (PMC13002419; doi:10.3389/fphar.2026.1787754)
Supplement: Supplementary file 1 [file Supplementaryfile1.docx]

Supplementary Material

Table S1. Antihypertensives Multum Lexicon therapeutic classification codes

| Therapeutic classification codes | Drug class |
| --- | --- |
| TC1S1 = 55 | Antihypertensive combinations |
| TC1S1 = 49 | Diuretics |
| TC1S1 = 42 | Angiotensin converting enzyme inhibitors |
| TC1S1 = 47 | Beta-adrenergic blocking agents |
| TC1S1 = 56 | Angiotensin II inhibitors |
| TC1S1 = 48 | Calcium channel blocking agents |
| TC1S1 = 53 | Vasodilators |
| TC1S1 = 44 | Antiadrenergic agents, centrally acting |
| TC1S1 = 43 | Antiadrenergic agents, peripherally acting |
| TC1S1_1 = 156 | Thiazide diuretics |
| TC1S1_1 = 475 | Potassium-sparing diuretics with thiazide |
| TC1S1_1 = 479 | Angiotensin II inhibitors with calcium ch |
| TC1S1_1 = 476 | ACE inhibitors with calcium channel blockers |
| TC1S1_1 = 473 | Angiotensin II inhibitors with thiazides |
| TC1S1_1 = 472 | Beta blockers with thiazides |
| TC1S1_1 = 470 | Miscellaneous antihypertensive combination |
| TC1S1_1 = 467 | ACE inhibitors with thiazides |
| TC1S1_1 = 154 | Loop diuretics |
| TC1S1_1 = 155 | Potassium-sparing diuretics |
| TC1S2 = 340 | Aldosterone receptor antagonists |


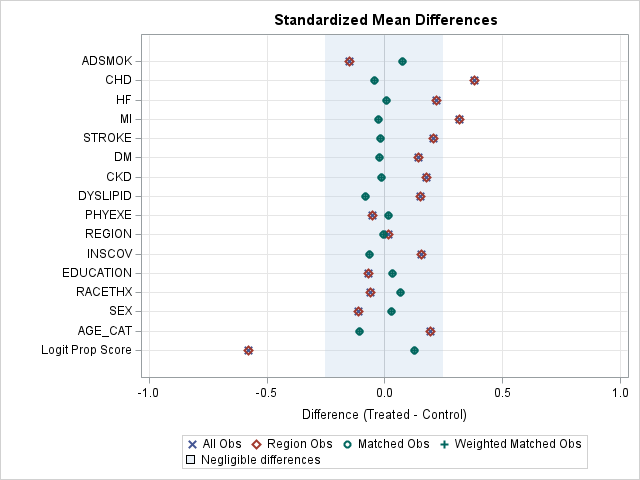


Figure S1. Standardized mean difference after propensity score matching


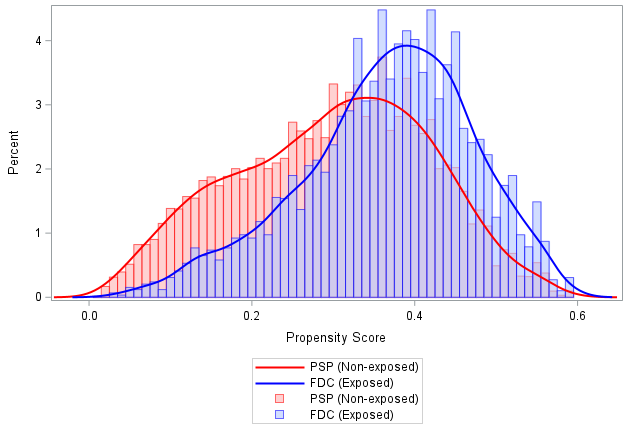


Figure S2. Distribution and overlap of propensity scores between treatment group. PSP = MPC (Multi-pill combination)

| **Outcomes** | **Univariate model** | | |
| --- | --- | --- | --- |
|  | RR | 95% CI | P-value |
| Office-Based Visits | 0.934 | (0.879 - 0.993) | 0.0281 |
| Outpatient Visits | 0.917 | (0.731 - 1.151) | 0.4552 |
| Prescription Fills | 0.853 | (0.815 - 0.893) | 0.0001 |
| Emergency Room Visits | 0.712 | (0.636 - 0.796) | 0.0001 |
| Hospitalization | 0.721 | (0.545 - 0.953) | 0.0219 |

Table S2. Results of the univariate model evaluating the association between FDC use versus MPC use and healthcare utilization
